# Butter Milk on Erysipelas

**Published:** 1914-04

**Authors:** 


BUTTER MILK ON ERYSIPELAS. Arnold, *London Pract.*, has treated all cases during the last 17 years with local applications of buttermilk, with good results.

